# Diet and Inflammation in Cognitive Ageing and Alzheimer’s Disease

**DOI:** 10.1007/s13668-019-0271-4

**Published:** 2019-04-04

**Authors:** Andrea M. McGrattan, Bernadette McGuinness, Michelle C. McKinley, Frank Kee, Peter Passmore, Jayne V. Woodside, Claire T. McEvoy

**Affiliations:** 1 Institute of Health and Society and Newcastle University Institute of Ageing, Newcastle University, Biomedical Research Building, Campus of Ageing and Vitality, Newcastle upon Tyne, NE4 5PL UK; 2 Centre for Public Health, Queen’s University Belfast, Grosvenor Road, Belfast, Northern Ireland BT12 6BJ UK

**Keywords:** Inflammation, Mechanisms, Pathways, Diet, Nutrients, Dietary patterns, Mediterranean diet (MD), Dietary approaches to stop hypertension (DASH), Pro-inflammatory diets, Cognition, Cognitive function, Cognitive ageing, Alzheimer’s disease

## Abstract

**Purpose of Review:**

Nutrition is known to modulate the immune system and may alter neuroinflammatory processes implicated in the pathogenesis of Alzheimer’s disease (AD) and progression of neurodegeneration. Here, we review the evidence for healthy dietary patterns and age-related cognition and discuss potential neuroinflammatory actions of diet on cognitive function.

**Recent Findings:**

Anti-inflammatory dietary patterns such as the Mediterranean diet (MD) and dietary approaches to stop hypertension (DASH) may be neuroprotective. Several dietary components consumed in the MD and DASH (omega-3 fatty acids, antioxidants and polyphenols) can inhibit neuroinflammation associated with AD. Anti-inflammatory diets may also attenuate neuroinflammation via indirect immune pathways from the gut microbiome and systemic circulation.

**Summary:**

Diet may influence cognitive ageing via several inflammatory pathways. However, data from human studies are lacking and the exact mechanisms linking diet to cognitive function remain elusive. Further dietary intervention studies are required to investigate diet-associated neurological change from the earliest through to latest stages of cognitive decline. Furthermore, incorporation of neuroimaging measures in intervention studies would advance current understanding of the mechanistic effects of dietary modification on neuroinflammation in the ageing brain.

## Introduction

Worldwide, 50 million people are living with Alzheimer’s disease (AD) and related dementias, and this figure is projected to triple by 2050 unless preventive measures are developed [1]. Addressing modifiable risk factors is considered to be the most promising strategy to prevent AD [1]. In this regard, increasing evidence suggests that dietary interventions have potential to protect against cognitive decline during ageing [2–4].

The mechanisms governing dietary influences on cognition are not clear, but inflammatory pathways are likely to be involved [5, 6]. Inflammation has been strongly implicated in the pathogenesis of AD [7••, 8]. Microglial macrophages in the brain become chronically activated during ageing [9], and particularly under pathological conditions [10], to promote sustained production of pro-inflammatory cytokines including interleukin-1β, interleukin-6 (IL-6) and tumour necrosis factor-α (TNF-α) [8, 11]. Production of these molecules can perpetuate a cycle of neuroinflammatory processes including amyloidosis, neuronal death [10, 12], cortical thinning [13, 14], reduced brain volume [13], cerebral vascular disease-related events such as microbleeds and infarcts [15, 16] and neurodegeneration [7••]. Genetic studies provide strong evidence for a causative role of neuroinflammation in AD, with several mutations in microglial genes, for example, TREM2 and CD33, being independently associated with elevated AD risk [17]. Furthermore, greater numbers of activated microglial cells and cytokines have been confirmed in brain tissue from individuals with AD [18].

Diet is known to modulate the immune system [19••], and several nutrients and bioactive components can influence neuroinflammatory processes in animals. For example, polyphenols, unsaturated fats and antioxidant vitamins inhibit oxidative stress and neuroinflammation [20, 21••, 22], while saturated fat promotes inflammation, particularly in the hypothalamus [23]. However, it is not clear whether diet-induced effects on neurocognition are mediated directly by neuroinflammatory processes and/or via other immune mechanisms in vivo. An increasing body of evidence suggests that peripheral inflammation and alterations to the gut microbiome can amplify neuroinflammation and accelerate neurodegeneration [7••, 24, 25] and these external factors can also be influenced by diet [26].

Mechanistic studies in the brain have primarily focused on single nutrients. However, the synergistic effects of nutrients and foods when consumed together as a usual dietary pattern are likely to exert greater effects on inflammatory processes and neurodegeneration during ageing [25, 27]. There has been much interest in examining the Mediterranean diet (MD) and dietary approaches to stop hypertension (DASH) dietary patterns as potential strategies for dementia prevention due to their proven anti-inflammatory [28–30] and cardioprotective effects [31, 32]. The MD and DASH have shown promising associations with slower rate of cognitive decline [33, 34] and reduced AD risk [35–38] although results have not been consistent [39, 40] and evidence for a causal relationship is limited due to the small number of diet intervention studies conducted. Interestingly, more recent neuroimaging studies have shown protective effects of MD on neuronal structures and early morphological changes linked to neurodegeneration and AD [41–48].

This review aims to summarise available evidence to evaluate the role of MD and DASH dietary patterns for cognitive health and AD risk during ageing and discuss underlying inflammatory mechanisms of diet on cognitive function in humans and animal models.

## Dietary Patterns and Cognitive Health During Ageing

### The Mediterranean Diet

The MD describes the traditional dietary pattern consumed in Mediterranean countries and is characterised by high intake of fruits, vegetables, wholegrains, nuts and legumes; moderate intake of fish, poultry and alcohol (particularly red wine, with meals) and low intake of red and processed meats with olive oil used as the main fat source [49, 50]. Adherence to the MD has demonstrated clear benefits for both primary [32] and secondary [51] CVD prevention. This may also be the case for cognitive function. A recent pooled analysis of available prospective data in older adults reported a beneficial association between adherence to the MD and cognitive function, especially for domains of global cognition and episodic memory [52].

Observational studies that have evaluated the MD for cognitive health are summarised in Table 1. In summary, greater concordance to the MD has been associated with better cognitive performance [53, 54, 57, 58, 65], slower rate of cognitive decline [61, 66–68] and reduced risk of cognitive impairment [53, 55, 56, 61] and AD [36]. However, the variable and sometimes disparate study findings [27, 59, 60, 62–64] are likely due to differences between studies in the populations investigated and methods used to assess diet and cognition. Most epidemiologic studies have examined MD adherence using a score system derived from population-specific median reported intake thresholds for each individual food component in the MD score and this approach limits the comparability of findings with other populations [53]. Furthermore, there is heterogeneity between studies with regards to the cognitive outcomes measured, follow-up time and population characteristics including baseline cognitive status.

**Table 1:** An overview of studies associated with anti-inflammatory dietary patterns

Study	Cohort details	Dietary pattern	Cognitive outcome measures	Key findings
Cross-sectional studies				
Cognitive impairment and dietary habits among elders: the Velestino Study [53]	*N* = 557 ≥ 65 years Greece	MD	MMSE < 24 or ≥ 24	MD adherence was positively associated with better MMSE score among men only. For women, an inverse association was detected.
Mediterranean diet and cognitive function: a French study [54]	*N* = 3083 ~ 52.0 years France	MD	Episodic memory, lexical- semantic memory, short term and working memory, mental flexibility	Low MD adherence was associated with poorer performance in one measure of attention, processing speed, and working memory.
Mediterranean diet and mild cognitive impairment [55]	*N* = 1875 (*N* = 1393 cognitively normal, *N* = 482 MCI) > 70 years USA	MD	Incidence of MCI and progression from MCI to AD.	Compared to those with the lowest MD adherence, those at the highest MD adherence had a 28% less risk of developing MCI and a 48% less risk of developing AD.
Adherence to a Mediterranean diet and Alzheimer’s disease risk in an Australian population [56]	*N* = 970 (723 healthy controls (HC), 98 MCI and 149 AD) > 70 years Australia	MD	Mini-Mental State Examination, Logical, Memory II (WMS; Story 1 only), California Verbal Learning Test II—Second Edition (long delay) Delis-Kaplan Executive Function System Verbal Fluency	One unit increase in MD score was associated with 13–19% lower odds of MCI, and 19–26% lower odds of AD.
Mediterranean diet and functional indicators among older adults in non-Mediterranean and Mediterranean countries [57]	*N* = 2791 US National Health and Nutrition Survey (NHANES) USA *N* = 1786 from Israeli National Health and Nutrition Survey (MABAT ZAHAV) Israel > 70 years	MD	NHANES - Wechsler adult intelligence scale, third edition digit symbol MABAT ZAHAV - MMSE	A greater MD adherence was associated with better cognitive function among both cohorts.
Neuroprotective diets are associated with better cognitive function: the health and retirement study [58]	*N* = 5907 > 60 years USA	MD	Global Cognition	Greater MD adherence was associated with better cognitive function.
Mediterranean diet and magnetic resonance imaging-assessed brain atrophy in cognitively normal individuals at risk for Alzheimer’s disease [45]	*N* = 52 ~ 54 years USA	MD	3D T1-weighted MRI scanning to provide estimates of cortical thickness for entorhinal Cortex (EC), inferior parietal lobe, middle temporal gyrus, orbitofrontal cortex (OFC) and posterior cingulate cortex (PCC)	MD adherence was associated with greater cortical thickness of AD-vulnerable regions.
Mediterranean diet and brain structure in a multi-ethnic elderly cohort [42]	*N* = 674 ~ 80 years USA	MD	Total brain volume (TBV), total grey matter volume (TGMV), total white matter volume (TWMV), mean cortical thickness (mCT), and regional volume or CT were derived from MRI scans	Compared to low MD adherence, higher MD adherence was associated with larger TBV, TGMV, and TWMV.
Mediterranean diet, micronutrients and macronutrients, and MRI measures of cortical thickness [48]	*N* = 672 ~ 80 years USA	MD	Magnetic resonance imaging measures of cortical thickness	Higher MD score was associated with larger frontal, parietal, occipital thickness and average lobar cortical thickness.
Longitudinal studies				
Adherence to a Mediterranean diet, cognitive decline, and risk of dementia [27]	*N* = 1410 ~ 76 years France	MD	Mini-Mental State Examination (MMSE), Isaacs Set Test (IST), Benton,Visual Retention Test (BVRT), and Free and Cued Selective Reminding Test (FCSRT), Incident cases of dementia	Higher MD score was associated with fewer MMSE errors, but not with other cognitive test scores. MD adherence was not associated with the risk for incident dementia.
Diet, physical activity and cognitive impairment among elders: the EPIC–Greece cohort (European Prospective Investigation into Cancer and Nutrition) [59]	*N* = 732 ≥ 60 years Greece	MD	Mini-Mental State Examination (MMSE)	Adherence to the MD was not associated with MMSE.
Vegetables, unsaturated fats, moderate alcohol intake, and mild cognitive impairment [60]	*N* = 1233 ≥ 70 years USA	MD	Incident MCI and dementia	Higher MD adherence was associated with lower risk of incident MCI or dementia.
Adherence to a Mediterranean diet and risk of incident cognitive impairment [61]	N = 17,478 ~ 64 years USA	MD	Incident cognitive impairment using a 6 item screen	Higher MD adherence was associated with less likelihood of incident cognitive impairment.
The Mediterranean diet is not related to cognitive change in a large prospective investigation: the PATH through life study [62]	*N* = 1528 60–64 years Australia	MD	Incident MCI	MD was not associated with cognitive decline.
Mediterranean diet habits in older individuals: Associations with cognitive functioning and brain volumes [63]	*N* = 194 ~ 75 years Sweden	MD	Seven minute screening (7MS) test; brain volume measured by volumetric magnetic resonance imaging	No associations between MD and 7MS nor volumes of grey matter and white matter in the fully adjusted models.
Mediterranean diet and cognitive decline in women with cardiovascular disease or risk factors [64]	*N* = 2504 (women) ≥ 65 years USA	MD	Telephone Interview of Cognitive Status (TICS), telephone adaptation of the Mini-Mental State Examination (MMSE). TICS 10-word list (immediate and delayed recalls) and the East Boston Memory Test (immediate and delayed recalls), Category fluency test.	MD was not related to cognitive decline.
Relation of DASH and Mediterranean-like dietary patterns to cognitive decline in older persons [33]	*N* = 826 ~ 82 years USA	MD DASH	Change in: Episodic memory, semantic memory, perceptual organisation, perceptual speed, working memory and global cognition	Higher MD score was associated with slower decline in episodic memory, semantic memory, working memory and global cognition. Higher DASH score was associated with slower rates of decline in episodic memory, semantic memory and global cognition.
Long-term adherence to the Mediterranean diet is associated with overall cognitive status, but not cognitive decline, in women [65]	*N* = 16,058 ≥ 70 years Women USA	MD	Change in Telephone Interview for Cognitive Status (TICs), verbal memory, global cognition	MD score was associated with better cognitive performance on tests but not with decline in TICs, or composite scores of verbal memory and global cognition.
Mediterranean diet and cognitive function: The SUN project [66]	*N* = 823 ~ 62 years Spain	MD	Cognitive function by Telephone Interview of Cognitive Status –modified (TICS-m)	Greater cognitive decline was observed among participants with low or moderate baseline adherence to the MD than among those with better adherence.
Dietary patterns and cognitive decline among Chinese older adults [67]	*N* = 1650 > 55 years China	MD	Repeated measures of global cognitive scores, composite cognitive z-scores (standardised units [SU]), and standardised verbal memory scores (SU).	Higher MD adherence was associated with a slower rate of cognitive decline.
Mediterranean lifestyle in relation to cognitive health: results from the HELIAD study [68]	*N* = 1716 ≥ 65 years Greece	MD	Diagnosis of Mild Cognitive Impairment (MCI), a composite Z-score for global cognitive functioning and Z-scores for 5 domains: memory, language, attention-speed of information processing, and executive and visual-spatial functioning	Greater MD adherence was associated with better cognitive performances in the domains of memory, visual- spatial functioning and language and composite cognitive Z –score.
Mediterranean diet and preserved brain structural connectivity in older subjects [46]	*N* = 146 ~ 73 years France	MD	3-T magnetic resonance imaging measuring Brain Grey matter and White matter volumes as well as a battery of neuropsychological tests	Greater MD adherence was significantly associated with preserved white matter microstructure in extensive brain areas.
Mediterranean diet is associated with slower rate of hippocampal atrophy: a longitudinal study in cognitively normal older adults [43]	*N* = 215 ~ 79 years USA	MD	Magnetic resonance imaging scans to assess change of hippocampal volumes (intracranial volume adjusted)	Higher MD adherence was associated with slower atrophy for total hippocampal volume, by approximately 2.5 years.
Mediterranean-type diet and brain structural change from 73 to 76 years in a Scottish cohort [44]	*N* = 562 72.65 ± 0.72 years UK	MD	Brain volume (total and grey matter) and cortical thickness using MRI imaging	Lower MD adherence was associated with greater 3-year reduction in total brain volume.
Mediterranean diet adherence and rate of cerebral Aβ-amyloid accumulation: data from the Australian Imaging, Biomarkers and Lifestyle Study of Ageing [47]	*N* = 77 ~ 71 years Australia	MD	Cerebral Aβ load using compound B positron emission tomography	Higher MD score was associated with less Aβ accumulation.
Prospective study of dietary approaches to stop hypertension and Mediterranean style dietary patterns and age-related cognitive change: the Cache County Study on Memory, Health and Ageing [69]	*N* = 3831 ≥ 65 years USA	MD DASH	Modified Mini-Mental State Examination (3MS)	Higher DASH and MD scores were associated with higher average 3MS scores. People in quintile 5 of DASH averaged 0.97 points higher than those in quintile 1. The corresponding difference for Mediterranean quintiles was 0.94.
No association between dietary patterns and risk for cognitive decline in older women with 9-year follow-up: data from the women’s health initiative memory study [70]	*N* = 6425 65–79 years USA	MD DASH	Cognitive decline was defined as cases of MCI or probable dementia (PD)	MD or DASH scores were not associated with MCI or PD.
Intervention studies				
Mediterranean diet improves cognition: the PREDIMED- NAVARRA randomised trial [2]	*N* = 522 74.6 ± 5.7 Spain	MD supplemented with olive oil or nuts vs low fat diet (control)	Global cognition measured by MMSE and Clock Drawing Test	Participants in the MD and olive oil and MD and nuts groups had higher mean cognitive scores than the control group.
Virgin olive oil supplementation and long-term cognition: the PREDIMED- NAVARRA randomised trial [71]	*N* = 285 74.1 ± 5.7 years Spain	MD supplemented with olive oil or nuts vs low fat diet (control)	Episodic memory, Verbal memory, Visual memory, Visuospatial abilities, Language Fluency, Executive function, Attention, Working memory, Abstract reasoning	The MD and olive oil group had a significantly better performance across fluency and memory compared to control. The MD and nuts group did not differ in cognitive performance in comparison to the control group.
Mediterranean diet and age-related cognitive decline: a randomised clinical trial [4]	*N* = 334 ~ 66.9 years Spain	MD supplemented with olive oil or nuts vs low fat diet (control)	Change in: Global cognition, Memory, Frontal (attention + executive function)	In comparison to the control, those in the MD and nuts group showed significant improvement in memory whereas those in the MD and olive oil group showed improvement in global cognition and frontal composite scores.
The Mediterranean diet and cognitive function among healthy older adults in a 6-month randomised controlled trial: the MedLey Study [72]	*N* = 137 72.1 ± 5.0 years Australia	MD vs habitual diet	Neuropsychological test battery, including 11 individual tests	Adherence to a MD had no significant effect on overall age-related cognitive performance.
Effect of the NU-AGE diet on cognitive functioning in older adults: a randomised controlled trial [73]	*N* = 1279 70.9 ± 3.4 years Five European centres in France, Italy, the Netherlands, Poland, and the United Kingdom (UK)	NU-AGE diet (individually tailored Mediterranean-like diet advice vs habitual diet (advice on national dietary guidelines)	Consortium to Establish a Registry for Alzheimer’s Disease (CERAD)-Neuropsychological Battery and five additional domain-specific single cognitive tests	Both control and intervention groups showed improvements in global cognition and in all cognitive domains after 1 year, but differences between the two groups were not statistically significant.
Effects of the dietary approaches to stop hypertension diet, exercise, and caloric restriction on neurocognition in overweight adults with high blood pressure [3]	*N* = 124 52.3 ± 9.6 years USA	DASH diet alone vs DASH combined with a behavioural weight management program including exercise and caloric restriction vs a usual diet control group.	Battery of neurocognitive tests to assess performance in the domains of executive function-memory-learning (EFML) and psychomotor speed	Compared to control, DASH combined with a behavioural weight management program improved EFML and psychomotor speed and DASH diet alone improved psychomotor speed.
Lifestyle and neurocognition in older adults with cognitive impairments: a randomised trial [74]	*N* = 160 65.4 ± 6.8 years USA	Aerobic exercise vs no exercise and DASH diet vs no DASH diet (health education)	Pre-specified composite measure of executive function; measures of language/verbal fluency, memory, and ratings on the modified Clinical Dementia Rating Scale	No effect of DASH diet on cognitive function. [74] The largest improvement in cognitive function was reported in response to combined exercise and DASH.

It is possible that MD contributes to neuronal integrity across the life-course. Emerging evidence from observational studies report a link between greater MD adherence and more favourable brain structures and functions that protect against neurodegeneration including increased cortical thickness [45, 48], greater brain volumes [42], slower rate of hippocampal atrophy and improved structural connectivity [43, 44, 46] as well as less amyloid (Aβ) accumulation at both midlife and older age [41, 47].

The effect of MD on neurocognition has been evaluated in a limited number of randomised controlled trials (RCTs). The PREDIMED study demonstrated a modest beneficial effect of increased MD adherence over 4–6 years on cognitive function in cognitively healthy adults at high CVD risk [2, 4, 71] particularly in domains of global cognition, memory and executive function [4]. In contrast, adopting a MD over 6 months had no effect on cognitive function in healthy older Australian adults [72]. The recent NU-AGE trial also reported no benefit of a Mediterranean-style diet on cognitive function after 1 year in older European adults; however, participants with greatest MD adherence demonstrated improved global cognition and episodic memory compared to those with low adherence [73].

### The Dietary Approaches to Stop Hypertension Diet

The DASH diet is an accepted non-pharmacological treatment for hypertension [31] and, like the MD, recommends a high intake of fruits, vegetables, nuts and wholegrain products. However, in contrast to a MD, DASH places greater emphasis on low fat dairy foods, low dietary sodium and does not recommend alcohol. In older adults, higher DASH scores have been associated with better cognitive function [69] and slower cognitive decline [33] but, again, findings have not been consistent [70]. There is limited evidence from intervention studies for an effect of DASH on neurocognition. The ENCORE study showed improved cognitive function in response to a calorie restricted DASH diet among overweight adults with hypertension [3], while preliminary 6-month data from the ENLIGHTEN study in cognitively impaired adults demonstrated no benefit of DASH diet alone on cognition, but improvement in executive function among those consuming DASH combined with aerobic exercise [74].

Collectively, the available evidence suggests that MD and DASH patterns may provide protection against neurodegeneration during ageing, with more consistent associations in favour of the MD, probably owing to a greater number of studies that have examined MD and cognition, relative to DASH or indeed other dietary patterns. Further prospective studies in diverse populations are recommended to determine relations between dietary patterns and clinically relevant measures of cognitive decline as well as incident AD. While few dietary intervention studies have been conducted, beneficial effects of MD on cognition are shown in trials of longer duration (12 months or more) [2, 4, 60] and in participants who adhere more closely to the intervention diet [63]. Further research is required to determine the duration of dietary intervention needed for optimal effect on cognition and to identify cognitive end-point measures that can respond to subtle diet-induced changes in the ageing brain. Furthermore, intervention strategies would benefit from learning how best to support behaviour change towards a healthy dietary pattern in different population groups.

The MD and DASH dietary patterns are typically low in saturated fat, sugar and high in antioxidants, fibres and polyphenols, which have potential independent effects on brain health. It is likely that the myriad of bioactive compounds and nutrients consumed at synergistic levels within these dietary patterns exert the potential to reduce neuroinflammatory processes involved in neurodegeneration [75].

## Inflammatory Mechanisms of Dietary Action on Cognitive Function

### Effects of Diet on Neuroinflammation

Nutrients have important physiological roles for normal brain functioning and are transported into the brain via the blood–brain barrier (BBB) or from the choroid plexus transport locus of the blood-cerebrospinal fluid barrier by distinct mechanisms, such as facilitated diffusion and active transport [76]. The effect of whole dietary patterns on neuroinflammation is yet to be discerned; however, animal studies have consistently reported anti-neuroinflammatory effects of several nutrients typically eaten in high amounts in MD and DASH diets. Antioxidants from fruit and vegetables suppress neuroinflammatory processes and neuronal apoptosis by inhibiting free radicals and cytokine production in activated microglia cells [22, 75, 77] and plant-derived flavonoids are thought to play a role in preventing neuroinflammation by downregulating transcription factor activity, e.g. NF-_K_B and inhibiting pro-inflammatory cell signalling pathways [78, 79]. Long chain omega-3 fatty acids from fish attenuate the expression of pro-inflammatory cytokines in microglia and help to resolve inflammation in the brain [20, 80].

While these animal studies provide important insights into mechanistic actions of diet on neuroinflammation, it is not clear how well the findings translate to humans, due to potential differences in nutrient bioavailability and metabolism. Indeed, single nutrient supplementation has shown no cognitive benefit among older adults in the few trials that have been conducted so far [81•, 82•], although positive signals have been observed for individuals with low baseline nutrient status [83] and for fish oil supplementation in early cognitive impairment [84], suggesting that population subgroups may derive cognitive benefit from nutrient supplementation.

### Indirect Inflammatory Actions of Diet on Neuroinflammation

#### Systemic Inflammation

Increased peripheral inflammatory markers have been associated with neurodegeneration [85] and are suggested to increase neuroinflammation via neuronal and hormonal pathways, as previously reviewed [7••, 19••]. The MD and DASH diet have demonstrated anti-inflammatory effects in humans. In a recent meta-analysis of six RCTs, DASH significantly decreased serum (high-sensitivity C-reactive protein (hs-CRP) concentration (mean difference − 1.01: 95% CI − 1.64, − 0.38; *I*^2^ = 67.7%) compared to usual diet which tended to be of greater magnitude in trials with longer duration [30]. The MD has been shown to decrease a broader range of inflammatory biomarkers. The PREDIMED study reported reductions in cytokines (IL-1, IL-6, IL-8, IL-12p70, CRP, TNF-α) and chemokines (MCP-1 and macrophage inflammatory proteins (MIP-1β)) in response to a 3-month MD supplemented with either olive oil or nuts, and the anti-inflammatory effects were observed up to 5 years of intervention [28, 29]. Adoption of a MD also significantly reduced hs-CRP, IL-6, IL-7, and IL-18 in patients with the metabolic syndrome [86]. Furthermore, key components of the MD, such as oily fish and omega 3 fatty acids, as well as bioactive polyphenols found in fruits and vegetables, red wine and olive oil have also been shown to reduce pro-inflammatory markers [78, 87, 88••]. Hence, the ability of dietary patterns, particularly the MD, to decrease systemic inflammation may help to attenuate neuroinflammation.

Emerging observational data lend support to systemic inflammation as a driver between diet and neurocognition (Table 2). High pro-inflammatory dietary scores have been linked with poor cognitive performance [91, 94] and cognitive impairment [92, 93]. Moreover, a proinflammatory dietary pattern, based on circulating IL-6 and characterised by higher intake of red meat, processed meat, peas and legumes, and fried food, and lower intake of whole grains has been associated with accelerated cognitive decline [89], while an inflammatory nutrient pattern derived from IL-6 and CRP levels and characterised by low intake of calcium, antioxidant vitamins, omega-3 and high intake of cholesterol, has been inversely associated with brain volume and cognitive function [90]. Further longitudinal studies are needed to evaluate whether dietary patterns based on systemic inflammatory biomarkers are related to incident AD.

**Table 2:** An overview of studies associated with pro-inflammatory dietary patterns

Study	Cohort details	Dietary pattern	Cognitive outcome measures	Key findings
Dietary pattern, inflammation and cognitive decline: The Whitehall II prospective cohort study [89]	*N* = 5083 56.0 years UK Longitudinal Study	Inflammatory dietary pattern characterised by higher intake of red meat, processed meat, peas and legumes, and fried food, and lower intake of whole grains which correlated with elevated IL-6 using reduced rank regression (RRR)	3 clinical examinations over 10 years were administered for the cognitive test battery and consisted of 4 standard tasks: Alice Heim 4-I, short-term verbal memory, phonemic fluency, and semantic fluency	A greater decline in reasoning was seen in participants in the highest tertile of adherence to the inflammatory dietary pattern compared to those in the lowest tertile after adjustment for confounding variables.
An Inflammation-related Nutrient Pattern is Associated with Both Brain and Cognitive Measures in a Multi-ethnic Elderly Population [90]	*N* = 330 79.0 years USA Cross Sectional Study	RRR was performed using 24 predetermined nutrients as predicting variables and two inflammatory biomarkers (CRP and IL6) as response variables. The inflammatory nutrient pattern (INP) was characterised by low intakes of calcium, vitamin D, vitamins E, A, B1, B2, B3, B5, B6, folate, omega-3 PUFA, and high intake of cholesterol	MRI imaging of global brain measures including intra-cranial volume (ICV), total brain volume (TBV), total grey matter volume (TGMV), and total white matter volume (TWMV). Cognitive ability at the time of MRI scan visit was measured with a neuropsychological battery	Each unit increase in inflammatory nutrient pattern was significantly associated with 36.8 cm3 smaller total brain volume and 0.21 lower visuospatial z-score.
Long-term association between the dietary inflammatory index and cognitive functioning: findings from the SU.VI.MAX study [91]	*N* = 3080 52.0 ± 4.6 years France	Dietary Inflammatory Index (DII) reflecting the overall inflammatory potential of the diet	Neuropsychological Evaluation including episodic memory, lexical–semantic memory, short-term and working memory	There was a strong inverse association observed between a higher DII (reflecting a more inflammatory diet) and overall cognitive functioning. With regard to specific cognitive domains, similar associations were observed with scores reflecting verbal memory, but not executive functioning.
Inflammatory potential of diet is associated with cognitive function in an older adult Korean population [92]	*N* = 239 74.0 years Korea	Energy adjusted dietary inflammatory index (E-DII) reflecting the overall inflammatory potential of the diet	Korean-adjusted version of the Mini-Mental State Examination (K-MMSE)	E-DII scores were significantly inversely associated with K-MMSE score in both unadjusted and adjusted models, after controlling for confounding variables. Participants in the highest E-DII tertile (reflecting a more inflammatory diet) had increased risk for mild or moderate cognitive impairment compared with those in the lowest E-DII tertile.
The association between an inflammatory diet and global cognitive function and incident dementia in older women: The Women’s Health Initiative Memory Study [93]	*N* = 7085 71.0 ± 3.9 years USA	Dietary Inflammatory Index (DII) reflecting the overall inflammatory potential of the diet	Cognitive function was evaluated annually, and MCI and all-cause dementia cases were adjudicated centrally	Higher DII scores (reflecting a more inflammatory diet) were associated with greater cognitive decline and earlier onset of cognitive impairment.
Dietary inflammatory index and memory function: population-based national sample of elderly Americans [94]	*N* = 1723 68.4 ± 0.2 years USA	Dietary Inflammatory Index (DII) reflecting the overall inflammatory potential of the diet	Consortium to Establish a Registry for Alzheimer’s disease (CERAD) Word Learning subset, the Animal Fluency test, and the Digit Symbol Substitution Test (DSST)	Episodic memory (CERAD), semantic-based memory (Animal Fluency Test) and executive function and working-memory (DSST) performances were lowest among those with the highest mean DII score (reflecting a more inflammatory diet).

#### The Gut Microbiome

The human gut microbiome represents the collective genomes of 10–100 trillion microorganisms harboured in the gastrointestinal tract and is considered important for healthy immune function [95]. The ageing process and other environmental factors can result in alterations to the microbiome composition (dysbiosis) and contribute to the development of chronic low-grade inflammation [96]. Dysbiosis stimulates excretion of endotoxins, e.g. lipopolysaccharides (LPSs) and microbial amyloids to promote permeability of the gut wall and increase peripheral circulation of proinflammatory cytokines [97]. Dybiosis has been implicated in the pathogenesis of AD by initiating and prolonging neuroinflammatory processes. Gut-derived bacteria and toxins can compromise the integrity of the BBB and contribute to early neuroinflammatory changes and AD by priming microglia and impairing amyloid clearance [24, 25]. Moreover, circulating LPSs and microbial amyloid activate innate resistance receptors, e.g. toll-like receptor (TLR) and receptor for advanced glycation end-products (RAGE) to amplify pro-inflammatory signalling and promote chronic neuroinflammation and progress neurodegeneration, particularly in brain regions sensitive to AD such as the hippocampus [25, 98].

Almost 60% of variation in the gut microbiome is attributable to diet [99]; therefore, modulating the gut microbiome through dietary means could be an effective approach to reduce inflammation associated with AD. However, only few studies have evaluated dietary patterns and gut microbiota. Preliminary data has shown positive associations between the MD and increased number of beneficial microbiota species, e.g. Bacteroidetes and their short chain fatty acid metabolites [100, 101] that have anti-inflammatory effects [26]. Further research is needed to understand the complex relationships between the gut microbiome and cognitive health and whether diet-induced effects on cognitive function are mediated by alterations in gut microbiota.

## Conclusion

Given the lack of effective treatments and projected increased prevalence of AD, there is considerable interest in understanding the contribution of neuroinflammation to the pathogenesis of AD in order to develop effective preventive strategies for cognitive decline.

Compelling evidence shows that nutrients and other bioactive dietary compounds influence neuroinflammatory processes leading to neurodegeneration in animals and that nutrients can act synergistically to exert greater biological effects. It is plausible that diets rich in anti-inflammatory components attenuate neuroinflammation via several immune pathways within the brain and indirectly from the gut microbiome and systemic circulation. However, data from human studies are lacking and the exact inflammatory mechanisms linking diet to cognitive function remain elusive.

Growing evidence supports a protective effect of anti-inflammatory dietary patterns, especially the MD, against cognitive decline in older persons but causal associations between diet and AD remain uncertain. The evidence base has been recently strengthened by small intervention studies showing improvements in cognitive function in response to MD and calorie-controlled DASH. However, adequately powered intervention studies with larger sample sizes and longer durations are required to examine the effect of dietary modification on clinically relevant cognitive outcomes. Neuroimaging studies have shown direct beneficial associations of MD on preclinical changes associated with AD; therefore, brain biomarkers should be considered as end-points in future intervention studies to investigate diet-associated neurological change from the earliest through to latest stages of cognitive decline. Furthermore, incorporation of PET (positron emission tomography) could allow measurement of in vivo microglia activation [102] and advance current understanding of the mechanistic effects of dietary modification on neuroinflammation in the ageing brain.

While most research attention has been given to MD and DASH, there is still much to learn about the ideal combination of foods and nutrients for optimal cognitive health during ageing. More recently, the MIND (Mediterranean-DASH diet intervention for neurodegeneration delay) dietary pattern that incorporates foods based on evidence in the diet-dementia field has shown to be more predictive of cognitive decline than the MD [103] and has also been associated with reduced AD risk [104]. The effect of MIND on cognitive decline is currently being evaluated in several US trials (e.g. clinicaltrials.gov Reference NCT02817074, NCT03688126) and will generate valuable data to determine whether improving diet quality is an effective strategy to improve brain health in older adults.
